# Bibliographical Department

**Published:** 1880-02

**Authors:** 


### BIBLIOGRAPHICAL DEPARTMENT.

"On the Early Delivery of the Placenta, when Previa." By Isaac E. Taylor, M. D. New York. This is a monograph of twenty-seven pages, reprinted from volume III. Gynecological Transactions for 1879. From the author. The pamphlet is neatly printed and the text illustrated with three well executed plates. It is written in Professor Taylor's usually graphic and pleasant style, and will amply repay perusal by any one.

"The Announcement of a Practical Treatise on Nervous Exhaustion. (Neuruosthenia.) Its Symptoms, Nature, Sequences and Treatment." By George M. Beard, M. D., of New York. This work is now in the press of Messrs. William Wood & Co., and will be issued this month. We venture to say, in advance, that it will be extensively read by the profession, as there are few, if any, better qualified for such an undertaking than Dr. Beard.

1. "A Report of the Results in Thirty-one Cases of Phthisis, at Aiken, S. C.; and, 2. Hints for Invalids Visiting Southern Health Resorts." By W. H. Geddings, M. D., of Aiken, S. C. The first pamphlet presents conclusive evidence of the value of the climate and surroundings of Aiken, as a resort for consumptives, and of the skillful treatment of Dr. Geddings. We know of no one better qualified for the care and professional management of such patients than Dr. Geddings, both by nature and education. The second brochure will be found valuable to the class indicated in the title, and is well and pleasantly written. From the author.

"The Second Annual Report of the Presbyterian Eye and Ear Charity Hospital of Baltimore, for the year ending December 31, 1879." This institution is under the government of a number of prominent ladies and gentlemen of this city, and from the report of J. J. Chisolm, M. D., surgeon in charge, seems to be doing a large amount of good. Dr. Chisolm says, "when we take into consideration the work done and the amount of relief given, we may well be satisfied with the retrospect."

"The Medical News and Abstract" is the title of a new monthly journal just issued by the enterprising publishing house of Henry C. Lea, of Philadelphia, and is under the editorial management of Dr. Minis Hayes. The January number is full of new and valuable matter to the profession, and we have no doubt will receive, as it merits, a large circulation. Subscription, $2.50 per annum.

"Chicago Medical Gazette." Number 2 of volume I. has been received, and we are pleased to place it on our list of exchanges. It is published on the 5th and 20th of each month. Dr. E. C. Dudley is the editor, aided by an editorial corps of seven gentlemen. The number before us is well filled with interesting and instructive reading matter. We wish it every success.

"Paquelin's Thermo-Cautery, with Wilson's Antithermic Shield, in Epithelioma of the Cervix Uteri." By H. P. C. Wilson, M. D., Baltimore, Md., Gynecologist to St. Vincent's Hospital and the Union Protestant Infirmary; Vice-President of the Baltimore Academy of Medicine, of the Medical and Chirurgical Faculty of Maryland, and ex-Vice-President of the American Gynecological Society, etc. The foregoing is the title of a sixteen-page pamphlet, embodying a paper recently read by the author before the Virginia Medical Society. Dr. Wilson's shield, spoken of in the text, promises to be a valuable adjunct to Paquelin's thermo-cautery apparatus. From the author.

"Gaillard's Medical Journal," formerly the "Richmond and Louisville Medical Journal." E. S. Gaillard, M. D., editor and publisher, 123 East Forty-sixth street, New York. Terms, $5 yearly, postage included. The February number of this well-edited and excellent journal has been received, and we cordially wish the editor and proprietor much success in his new field of labor.

"The Medical Record." A weekly journal of medicine and surgery, published by Wm. Wood & Co., and edited by George F. Shrady, M. D. Subscription, $5 per annum. This valuable and widely circulated journal is too well and favorably known to need any commendation from us. It is gladly placed on our list of exchanges.

"The Philadelphia Medical Times," a bi-weekly journal of medical and surgical science. Edited by Prof. Horatio C. Wood, M. D. Subscription, $4 per annum, in advance. Published by J. B. Lippincott & Co. Has also been received in exchange. There are few more zealous workers in the profession than Dr. Wood, and he manages always to make the "Times" equal to the requirements of its readers, notwithstanding his other duties.

"Louisville Medical News," a weekly journal of medicine and surgery. Edited by Richard O. Cowling, A. M., M. D., and Lunsford P. Yandell, M. D. Issued every Saturday. $3 a year, in advance. Is a valuable weekly, and fully abreast of the times.

"The Cincinnati Medical News." Edited by J. A. Thacker, A. M., M. D. $2 a year, in advance. Is an old and well-established journal, too well known to need commendation from us.

"The Ohio Medical Recorder." Edited by J. W. Hamilton, M. D., and J. F. Baldwin, M. D. Is another journal from the same State, of deserved reputation in the profession.

"The Canada Medical and Surgical Journal." Edited by George Ross, A. M., M. D., and W. A. Molson, M. D., M. R. C. S., England, and published in Montreal. Terms, $3 per annum, in advance. An excellent monthly.

"Maryland Medical Journal." Baltimore. Editors: H. E. T. Manning, M. D., T. A. Ashby, M. D. January, 1880. $3 a year, in advance. We cheerfully place this excellent journal upon our exchange list, and cordially extend the right hand of editorial friendship to its enterprising editors.

"The Virginia Medical Monthly." Landon B. Edwards, M. D., editor and proprietor, Richmond. Terms, $3 per annum, in advance. Single copies 30 cents. Though mentioned last, it is far from being the least in value and importance. In fact, we think it one of the best of the three dollar monthlies.

WE HAVE received and gladly add to our exchange list the following dental journals: The "Dental Register," the "Missouri Dental Journal," the "Dental Cosmos," "Johnston's Dental Miscellany," and the "American Journal of Dental Science," all of which are well known as the leading dental periodicals of this country. They are published monthly at $2.50 each per annum.

We have also received two favorably known quarterly publications, the "Dental Advertiser," and the "Dental Office and Laboratory."

We welcome the "Scientific American" every Friday.

"Correspondenz Blatt für Zahnärzte," Berlin, herausgegeben by C. Ash, has just been received, and is the first happy tidings that the PRACTITIONER is acknowledged in Europe.

"How to Keep Teeth Clean and Healthful." By George A. Mills, of Brooklyn, N. Y. This well-written article deserves the attention of the medical and dental profession.

We have received a copy of the "Dental Headlight," (Vol. I., No. I.,) a quarterly sheet, edited by Herman & Morrison Bros., of Nashville, Tenn.

The above-mentioned are the exchanges with which the PRACTITIONER has thus far been honored.

THE NUMBER of copies of the first issue of the PRACTITIONER that was returned in accordance with our Special Notice, by those who had not already subscribed, was very small. This shows conclusively that our efforts have been appreciated by the intelligent readers in the profession to whom they were sent. We are happy to say that our subscription list has outgrown our most sanguine hopes. For this reason we will increase our February issue to 5,000, and we hope our circulation during the year 1880 will be 10,000 copies.

"Placenta Previa; Outline of its Present Management, of a New Treatment, with an Illustrative Case." By R. J. Nunn, M. D., Professor of Theory and Practice of Medicine in the Savannah Medical College. The above monograph has just been received from our old friend, the author, but too late for further notice in the present issue.

WE make no apology for occupying so large a portion of the original department of the PRACTITIONER, and the consequent omission of other articles in this number of the journal, by publishing Professor Taylor's valuable paper entire. Dr. Taylor has more than an American reputation in the department of the profession of which his paper treats. From the days of his pupilage, under the great Cazeaux, of Paris, few men have cultivated that branch as assiduously and successfully as he has done, and it is no disparagement to others to say his peers are few in it in any country.

